# Is the EnodePro^®^ a Valid Tool to Determine the Bar Velocity in the Bench Press and Barbell Back Squat? A Comparative Analysis

**DOI:** 10.3390/s25020549

**Published:** 2025-01-18

**Authors:** Nina Behrmann, Martin Hillebrecht, José Afonso, Konstantin Warneke

**Affiliations:** 1 Institute of Sport Science, Carl von Ossietzky University of Oldenburg, 26129 Oldenburg, Germany; nina.behrmann@uni-oldenburg.de; 2 University Sport Center, Carl von Ossietzky University of Oldenburg, 26129 Oldenburg, Germany; hillebre@uni-oldenburg.de; 3 Centre of Research, Education, Innovation, and Intervention in Sport (CIFI_2_D), Faculty of Sport, University of Porto, 4200-450 Porto, Portugal; jneves@fade.up.pt; 4 Institute of Human Movement Science, Sport and Health, University of Graz, 8010 Graz, Austria; 5 Institute of Psychology, Leuphana University Lüneburg, 21335 Lüneburg, Germany; 6 Department for Human Movement and Exercise Physiology, Friedrich Schiller University Jena, 07743 Jena, Germany

**Keywords:** EnodePro, velocity-based training, bar velocity, bench press, deep squat, 1RM, measurement error, validity

## Abstract

In recent years, the EnodePro^®^ device has been one of the most frequently used velocity sensors to track the bar velocity in resistance training, with the aim of providing load–velocity profiles. However, recent articles highlight a lack of reliability and validity in the estimated maximal strength, which can cause a serious health risk due to the overestimation of the bar velocity. With this study, we aimed to investigate whether imprecision in the measurement could explain the variance in this measurement error. Methods: The research question was evaluated by comparing the integrated velocities from the EnodePro^®^ with the velocities from a high-resolution displacement sensor for the squat and bench press. The velocity was measured with loads corresponding to 30%, 50%, and 70% of the one-repetition maximum (1RM) in moderately trained participants (n = 53, f = 16, m = 37). Intraclass correlation coefficients (ICC) for agreement were supplemented by an exploration of the systematic bias and the random error (mean absolute error (MAE), mean absolute percentage error (MAPE)). Results: The results indicated movement specificity, with the ICC values for the squat ranging from 0.204 to 0.991 and with ICC = 0.678–0.991 for the bench press. Systematically higher velocities were reported by the EnodePro^®^ sensor (*p* < 0.001–0.176), with an MAE = 0.036–0.198 m/s, which corresponds to an MAPE of 4.09–42.15%. Discussion: The EnodePro^®^ seems to provide overly high velocities, which could result in the previously reported overestimation of the 1RM. Despite the validity problems of force/load–velocity profiles, we suggest evaluating the bar velocity with accurate measurement devices, which is, contrary to previous reports, not the case with the EnodePro^®^.

## 1. Introduction

The accurate, precise and valid determination of performance capacities is paramount in sports medicine and exercise science, especially when aiming to measure training effects and adjusting exercise routines [1] While the one repetition maximum (1RM) test is probably the most common way to determine the strength capacity in athletes reliably [2], the literature outlines several practical limitations, such as time-consuming testing protocols that require extensive warm-up phases, as well as supervision, which complicates their implementation in group settings [3]. To counteract these limitations of the percentage-based training intensity regulation and maximal strength diagnostics via the 1RM (such as daily form variability [4] or injury risk in group settings [3,5,6]), velocity-based load estimations have been suggested as viable alternatives [7,8,9].

Controlling the load via percentage-based approaches requires the direct testing of the 1RM [10], while velocity-based approaches assume an inverse linear relationship between the used (submaximal) training loads and the bar velocity [11]. By performing submaximal trials (e.g., 30%, 50% and 70% of the 1RM [12]), the dynamic maximal strength is estimated by assuming target velocities corresponding to 100% of the 1RM. Without testing for 1RM, assuming a velocity of 0 m/s would correspond to the maximal strength (action = reaction) served to estimate the load of the 1RM [13]. In dynamic testing, the velocity is never v = 0, and “minimal velocity approaches” have found their way into practice to estimate the 1RM and subsequently control the training load based on these estimates [14].

The mandatory prerequisite for this procedure is the very precise, reliable and valid monitoring of the velocity via velocity sensors. Several velocity measurement devices exist, categorized as isoinertial dynamometers, linear velocity transducers (attached to a cable–pulley system connected to the barbell [2,15]), optical motion capture systems [16,17] and inertial measurement units (IMUs) [18]. Due to their user friendliness (space saving, immediate data access, mostly app-based data processing), IMUs are commonly used in practice and research [19,20], with the EnodePro (also known as the VMaxPro) as one frequently used wireless velocity tracker [16,21,22].

Although previous articles have critically questioned the validity of inverse linear relationships [13,23], this discussion seems obsolete if sensors do not fulfill the precision and reliability requirements to accurately measure the bar velocity. In turn, if the bar velocity is tracked insufficiently or without the required precision, further load–velocity relationships must be considered invalid, inhibiting their integration into practical load control procedures. Previous studies have compared the velocities measured via the EnodePro with those of other velocity sensors [23,24] or those from a 3D MoCap. Although studies have highlighted strong agreement (mean difference: −0.014 [95% CI −0.057–0.029], r^2^ = 0.99 [21,22,25]) and satisfying between-session reliability [26], these evaluations have limitations. First, validation via other (wireless) devices with unknown accuracy raises questions about the validity of the comparisons. Second, statistical concerns stem from the confusion of correlation-based statistics and agreement analyses. The relevance of distinguishing between agreement and relationship analyses was outlined in as early as 1989 by Lin [27], while the most common way to assess the agreement between devices is Bland–Altman analysis (BA). Nevertheless, the limits of agreement provide the sole quantification of a range including 95% of the differences between the devices, depending on the mean of both values [28,29]. To quantify the mean of the absolute measurement errors, while simultaneously presenting a unit-less measurement error value without the limitations of the ICC [30,31], studies have adopted the mean absolute and mean absolute percentage errors (MAE and MAPE) [1,23].

Given the relevance of assessing the actual velocity of the bar, the aim of this investigation was to assess the agreement within the EnodePro, a high-resolution linear displacement sensor, at velocities with different loads (30%, 50%,70% of 1RM [12,32]), as previously used in the literature. The evaluation was performed based on criticism of the previous statistical methods stated above, and it assessed device agreement using the concordance correlation coefficient (CCC) and Bland–Altman analysis [28,29]. Additionally, to check whether one sensor systematically yielded higher or lower velocities, the systematic bias was assessed, while the mean absolute error (MAE) and the mean absolute percentage error (MAPE) accounted for random errors between the devices [33]. Based on previous research [22,34], the device and testing movement specificity was hypothesized.

## 2. Materials and Methods

### 2.1. Experimental Approach to the Problem

To ensure the correct determination of the bar velocity, a Micro-Epsilon displacement sensor with accuracy of <0.15 mm (reported by the manufacturer) was considered as a sufficient device to determine the velocity by using the displacement per time (both can be measured with high precision). The agreement between this manual sensor and the EnodePro sensor was explored by performing the bench press and deep squat exercises, as two very common exercises [14]. To validate the EnodePro, the agreement between the displacement sensor and the remote Enode sensor was established by attaching both sensors to the same bar in a Smith machine to determine the velocity within the exact same movement.

### 2.2. Participants

Due to its exploratory nature, we used previous similar studies to determine a reasonable sample size for this study. G-Power does not provide the possibility to select agreement analyses for sample size calculations; therefore, we doubled the sample size of the Dragutinovic et al. [22] study, which provided a similar research design and question by seeking a comparison of the velocities measured via 3D motion capture and the EnodePro device. Therefore, a sample size of 53 participants was included in our study, which was justified by a post-hoc power analysis. Participants who were recreationally active (16 female, age: 24.29 ± 2.23 years; height: 171.64 ± 6.26 cm, mass: 63.57 ± 4.07 kg; and 37 male, age: 26.52 ± 3.97 years, height: 184.44 ± 6.59 cm, mass: 85.22 ± 9.73 kg) were included in the study. The participants were mostly physical education and kinesiology students in the graduate program of the university and were therefore considered recreationally active and participated at least twice per week in a structured exercise and training program. To counteract potential limitations due to the unfamiliar testing conditions, which could bias the validity of the tests [23], participants were included if they regularly performed the bench press and squat movements in their training. Furthermore, the participants had to have experience with the squat (at least parallel) and with bench pressing to avoid unfamiliar testing conditions. Participants were excluded from participation in the study if they had experienced a serious injury (defined as injuries that caused an immobilization phase or reduced physical activity level) within the previous six months, as well as excluding underaged participants (<18 years of age) or older participants. Due to incorrect movement execution, 4 participants were unable to perform a deep squat (values excluded from data processing), which reduced the sample to 49 participants (15 women; 34 men).

The study protocol was conducted in adherence to the Declaration of Helsinki. All participants provided written informed consent, and the study procedure was approved by the Ethics Committee of the University of Oldenburg (Drs.EK/2024/047).

### 2.3. Testing Protocol

**Figure 1:**
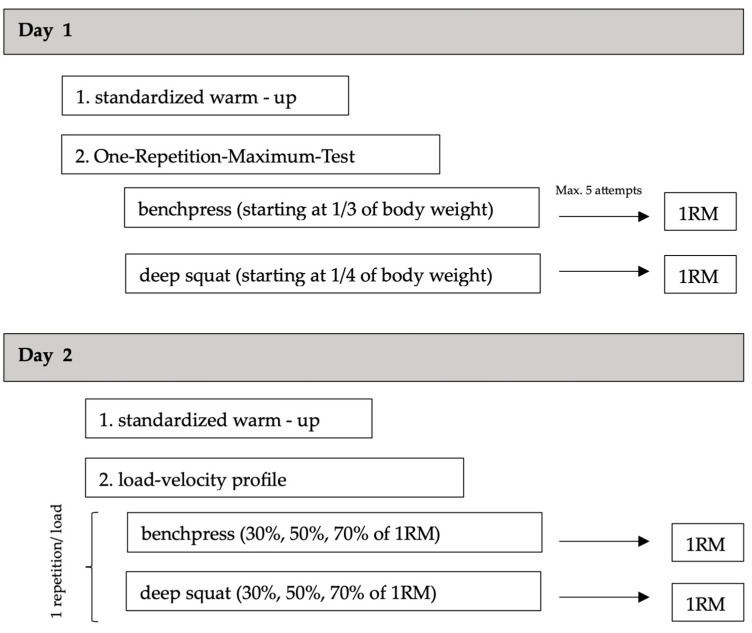
Schematic representation of the experimental procedure of the two test days.

The participants visited the lab twice, with a wash-out period of at least three days and a maximum of seven days. On both testing days, the participants performed a standardized warm-up protocol, performing light jogging for approximately 5 min with perceived exhaustion of between 6 and 8 on a scale ranging from 0 to 14 (adjusted BORG scale) [35]. The first testing session was used to introduce the participants to the testing procedure and to determine the dynamic maximum strength via the 1RM in the bench press and deep squat. The results were used to determine the loads used on the second testing day to assess the load–velocity relationship. Based on the results from Soriano et al. [36], Spitz et al. [12] and Martinez-Cava et al. [32], we used 30%, 50% and 70% of the 1RM. Detailed testing information is provided in the following sections. Figure 1 provides an overview of the experimental protocol.

### 2.4. One Repetition Maximum Test

The one repetition maximum test (1RM test) is a standard method used to determine the maximal dynamic concentric strength [2]. The objective of this test was to perform a single repetition with the highest possible weight while ensuring a correct technique and exercise execution, which was supervised by the investigators. In both movements—the squat and the bench press—a short rest period was ensured at the end of the eccentric phase to avoid bouncing movements in the lowest position.

During the preliminary test, the load started at approximately one-third of the body weight for the bench press measurement and one-quarter for the squat measurement. These loads were established as starting weights, as they represented light to moderate initial loads, allowing the participants to perform the movements in a controlled manner and with a proper technique. Furthermore, due to the varying training levels of the participants, this approach enabled standardization across the participants, regardless of their training experience, gender and performance level. The load was progressively increased until reaching the 1RM. To avoid fatiguing effects, a maximum of five attempts were performed. If the participant was not able to reach their respective considered maximal force output within this session, the test was finalized on the subsequent day. As a warm-up, the participants were instructed to perform the standardized warm-up and afterwards use their typical warm-up protocol to reach the 1RM. For the warm-up with submaximal trials, the inter-repetition rest was standardized at 1 min. As soon as moderate and high loads were reached [37], the recovery between each trial was extended to 2 min to avoid fatigue.

#### 2.4.1. Bench Press Measurement

**Figure 2:**
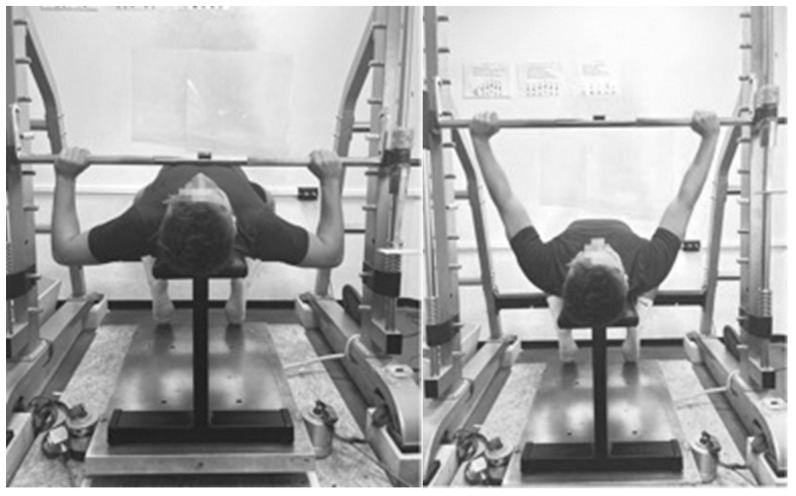
Representation of the experimental setup and the starting position (**left**) and ending position (**right**) of the concentric bench press movement.

During the bench press measurement, the participants adopted a lying position on a bench with their feet planted on the ground (see Figure 2). A slight arch was permitted in the lower back as long as the hips remained on the bench. The participants were instructed to use their typical grip width to avoid unfamiliar pressing conditions. The eccentric exercise was performed in a controlled manner for a maximum of 1 s to avoid fatigue. As soon as the bar reached the chest, a rest period of at least one second was ensured by the investigator, before the participant was instructed to press the bar as quickly as possible upwards until the elbows reached an extended position. As a maximum, two trials were allowed.

#### 2.4.2. Squat Measurement

**Figure 3:**
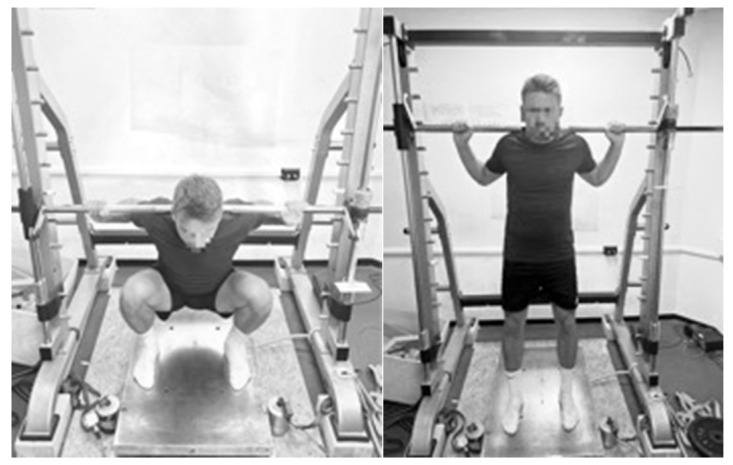
Starting position (**left**) and ending position (**right**) of the deep squat for measurement of the concentric phase.

For the deep squat measurement, the participants stood upright on the force plate in a self-selected foot position, with an 11.5 kg barbell positioned on the ascending trapezius muscle (see Figure 3). The required depth was achieved when the hips were below knee level, so that the thighs moved beyond parallel to the floor and the thigh touched the calf. Correct movement execution was ensured by two investigators independently supervising the squat. Furthermore, a butt wink and knee or ankle values were not permitted. Squat attempts were considered unsuccessful if the participant could not stabilize the bar with their back, lost the bar or did not reach the required depth. The participants were accompanied by a spotter. As a maximum, two trials were allowed.

### 2.5. Load–Velocity Profile

After the standardized warm-up, which was the same as that used during the 1RM testing, the load–velocity profile was measured with loads corresponding to 30%, 50% and 70% of the previously determined 1RM. On the one hand, these load levels were selected because they represented different intensity ranges (low, medium, high) that are commonly used in resistance training [12]. On the other hand, the use of three loads [32] allowed for adherence to a time-efficient protocol, minimizing fatigue in the test participants. The rest interval between each trial was again at least 2 min. The participants were instructed to exert the maximum force and speed during the concentric phase of the bench press and squat movements, while maintaining the same movement pattern as during the 1RM test. A brief rest period of approximately 1 s was implemented after the eccentric muscle action and before the concentric muscle action to prevent coupling between the eccentric and concentric movements.

### 2.6. Materials

**Figure 4:**
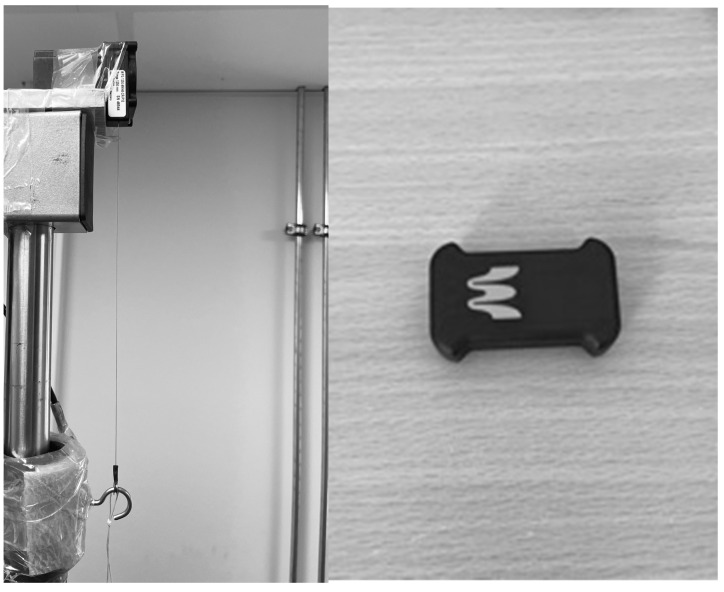
The Micro-Epsilon WPS-1250-MK46-CR-P10 displacement sensor, attached to the side of the barbell, and the EnodePro sensor, which was magnetically positioned in the center of the barbell during the measurement.

The load–velocity profile was monitored using two mobile systems: a wireless inertial measurement unit (EnodePro sensor, Blaumann & Meyer Sports Technology UG, Magdeburg, Germany) with a sampling rate of 200 Hertz and a displacement sensor, model WPS-1250-MK46-CR-P10 (Micro-Epsilon, Messtechnik GmbH & Co., KG, Ortenburg, Germany), which tracked the displacement during the movement. This sensor (left picture in Figure 4) operates based on variable resistance (potentiometer). A wiper moves along a resistance strip as the object being measured moves. The resistance changes proportionally to the wiper’s position, and this change is converted into an electrical voltage, which is then measured and is directly proportional to the object’s position. The voltage is transmitted through a DMS GEN5 preamplifier to an A/D converter (National Instruments DAQ 700, Austin, TX, USA), where U is digitized. The number of bits determines the accuracy of the measurement: the higher the bit count, the finer the resolution of the force plate. In this study, an A/D converter with a 13-bit resolution was used, capturing n = 2^13^ = 8192 values. The converted signal could be recorded via a computer. Since only the vertical axis was measured, a single-channel signal was considered, which could be analyzed using an individual measurement program. The sampling was performed at a frequency of f = 1000 Hz. Values were recorded with accuracy of <0.15 mm (Micro-Epsilon, Messtechnik GmbH & Co., KG, Ortenburg, Germany). Essentially, the sensor was used to continuously measure the displacement, and, from these data, the velocity was determined by calculating the rate of change in the position (displacement) of the object over time. This manual sensor was considered as the gold standard, as it appears to be impossible to track the individual components of the velocity (way and time) with higher accuracy.

Following the manufacturer’s instructions, the EnodePro sensor was positioned between the hands and the ends of the barbell, while the displacement sensor was attached to one end of the barbell via a cable. The data obtained from the EnodePro were transmitted via Bluetooth and analyzed using custom software, the Enode App Version 2.1.1. The mean and maximum velocities of all trials were manually recorded and compiled in a Microsoft Excel spreadsheet (Microsoft Corporation, Redmond, Washington, DC, USA).

### 2.7. Data Processing and Statistical Analysis

Average velocities were calculated based on the velocity curve, starting when the movement exceeded 0.1 m/s and ending when it fell below 0.1 m/s. Each category is presented as the mean (M) ± standard deviation (SD); maximum (Vmax) and mean (Vmean) velocities were determined by averaging all recorded values. Subsequently, the Vmax and Vmean were grouped according to the loads corresponding to 30%, 50% and 70% of the 1RM as, e.g., V30max, V50max [22].

A normal distribution was ensured using the Shapiro–Wilk test. The subsequent data analysis was conducted using JAMOVI (Version 2.5.7) with the *regression*, *seolmatrix*, *blandr* and *SimpleAgree* packages. First, the linear relationship between the variables was calculated using a Pearson correlation with

(1) $$r=\frac{\sum_{i=1}^{n}\left(x_{i}-\bar{x})(y_{i}-\bar{y}\right)}{\sqrt{\sum_{i=1}^{n}{\left(x_{i}-\bar{x}\right)}^{2}}\cdot \sqrt{\sum_{i=1}^{n}{\left(y_{i}-\bar{y}\right)}^{2}}},$$

where x_i_ and y_i_ = individual values of the two variables and $\bar{x}$ and $\bar{y}$ = mean values of the variables. ∑ denotes the sum over all values. The intra-day reliability was assessed through relative reliability analysis using ICCs for agreement and the concordance correlation coefficient (CCC) [27]:

(2) $$\rho_{c}=\frac{2\cdot r\cdot SD_{x}\cdot SD_{y}}{{SD}_{x}^{2}+SD_{y}^{2}+{\left(x-y\right)}^{2}},$$

where r is the Pearson correlation coefficient between the two datasets, and SD_x_ and SD_y_ are the standard deviations of datasets x and y, respectively.

To assess the absolute agreement among the specific raters in this study, the ICC two-way mixed measure was calculated as

(3) $$\mathrm{ICC}=\frac{\left({MS}_{R}-{MS}_{E}\right)}{\frac{{MS}_{R}+({MS}_{C}-{MS}_{E})}{n}},$$

where MS_R_ = mean square for rows (subjects/participants), MS_E_ = mean square for error, MS_C_ = mean square for columns and n = number of subjects. Additionally, a concordance analysis was performed using Bland–Altman (BA) plots [29], where the lower and upper limits of agreement, as well as the systematic bias (mean differences between the two sensors during the same trial), were quantified.

The qualitative inspection of random scattering was conducted by calculating the mean absolute error (MAE) [33,38], with

(4) $$MAE=\frac{1}{n}\cdot \sum_{i=1}^{n}\left|x_{i}-y_{i}\right|$$

as well as the mean percentage error (MPE),

(5) $$MPE=\frac{1}{n}\cdot \sum_{i=1}^{n}\frac{x_{i}-y_{i}}{x_{i}}\cdot 100$$

and the mean absolute percentage error (MAPE) [39,40], with

(6) $$MAPE=\frac{1}{n}\cdot \sum_{i=1}^{n}\left|\frac{x_{i}-y_{i}}{x_{i}}\right|\cdot 100$$

where the displacement sensor WPS was used as the reference. N indicates the total number of values in the test set, x_i_ denotes the individual values from the displacement sensor WPS and y_i_ denotes those from the EnodePro. To assess the precision and quantify the measurement uncertainties, the standard error of measurement (SEM) was additionally reported, and the minimal detectable change (MDC) for a 95% confidence interval was derived from it. The MDC indicates whether a change is significant and not due to random errors.

(7) $$SEM=SD\cdot \sqrt{1-ICC}$$

(8) $$MDC=SEM\cdot \sqrt{2\;}\cdot 1.96$$

Values exceeding three times the standard deviation were classified as potential outliers. A separate analysis that includes the outliers is provided in Appendix A. To evaluate their influence on the results, a sensitivity analysis was performed, excluding such values in addition. A *t*-test was used to check for systematic errors because it could test whether the mean of the differences (Mean Diff.) between the measurements of the two sensors significantly deviated from zero. In this context, the systematic error was defined as a constant deviation between the two devices. Since both sensors were attached to the same barbell (albeit at different positions) and measured the same trial, it was assumed that both would provide the same velocity. Deviations were calculated using the CCC and BA analysis, as well as the MAE and MAPE.

## 3. Results

The normal distribution of the data was ensured with *p* > 0.05. The average velocities ranged from 0.48 ± 0.12 m/s to 1.63 ± 0.35 m/s. Table 1 presents the average maximum and mean velocities for the bench press and squat measurements across all values. Table 2 (V30max, V50max, V70max) and Table 3 (V30mean, V50mean, V70mean) present the maximum and mean velocities for the respective loads based on the 1RM. Descriptives are provided as means (M) and standard deviations (SD). The agreement analysis, including the MAE, MAPE, MPE and LoAs, is provided for each exercise separately.

### 3.1. Agreement Between the Two Velocities

The correlation coefficients for all results ranged between r = 0.445 and r = 0.991 between the two devices. While the correlation coefficients for the bench press ranged between 0.882 and 0.979, the range was considerably larger for the squat measurements. In the squat, the correlation values fell between 0.445 and 0.945, with stronger correlations observed in the higher velocity ranges (except V70mean).

The presentation of the results for the different loads as a percentage of the 1RM is provided separately for the bench press and squat measurements.

#### 3.1.1. Bench Press Measurement

The ICC values ranged from 0.904 (for V30mean) to 0.987 (for V50max), with CCC values between 0.822 and 0.973 for the bench press movement.

The average velocities ranged from 0.50 ± 0.14 m/s to 1.63 ± 0.35 m/s (for the EnodePro) and 0.48 ± 0.12 m/s to 1.59 ± 0.32 (for the WPS). The random error was quantified via MAEs of 0.036 m/s to 0.073 m/s, which corresponded to an MAPE of 4.09% to 8.79%. In all measurements, except for the V70max, there was significant systematic bias (*p* < 0.001–0.025) (see Table 2 and Table 3). The MPE between the velocity measurement devices ranged from −8.36% to 8.36%.

**Figure 5:**
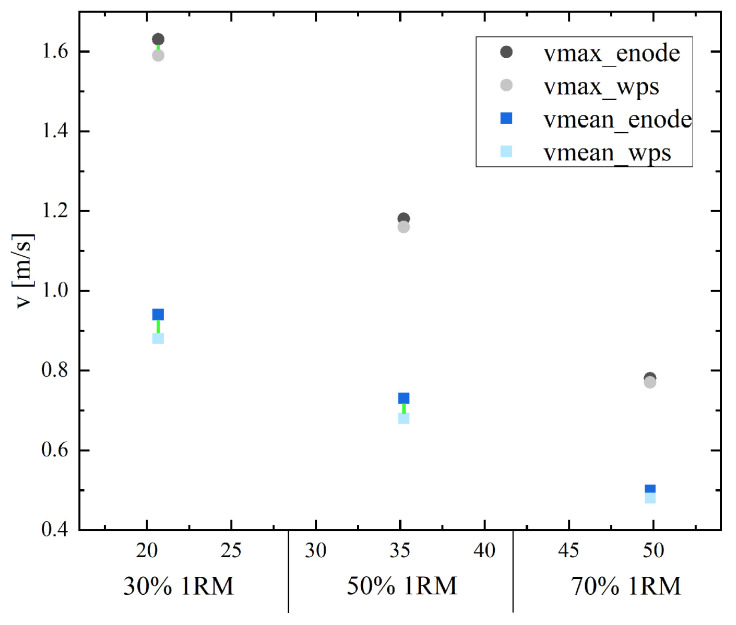
Maximum and mean velocities for the load at 30%, 50% and 70% of the 1RM for both sensors (the EnodePro and the WPS displacement sensor) during the bench press measurement. The connecting line represents the size of the MAPE: MAPE < 10% green line, MAPE 10–20% orange line, MAPE > 20% red line.

Figure 5 presents the maximum and mean velocities for the load at 30%, 50% and 70% of the 1RM from both sensors. The connecting line between the points classifies the MAPE.

#### 3.1.2. Deep Squat Measurement

For the squat measurement, the ICCs for the agreement ranged from 0.580 (for V30mean) to 0.900 (for V30max), with CCC values between 0.403 and 0.815.

The average velocities showed values between 0.59 ± 0.16 m/s and 1.49 ± 0.24 m/s (for the EnodePro) and 0.54 ± 0.10 m/s and 1.36 ± 0.23 m/s (for the WPS). The MAEs of 0.076 m/s to 0.123 m/s corresponded to an MAPE of 9.99% to 42.14%. All measurements exhibited systematic bias (*p* < 0.001–0.007) (see Table 2 and Table 3). Consistently, the EnodePro overestimated the velocities measured by the linear position sensor. The MPE between the velocity measurement devices ranged from −36.18% to −7.44%. The maximum error between the EnodePro and the WPS linear position sensor was observed for the V30mean. The second-highest MPE still showed a discrepancy of 18.19% between the two velocity measurement devices at the lowest velocity (V70max) for the deep squat.

**Figure 6:**
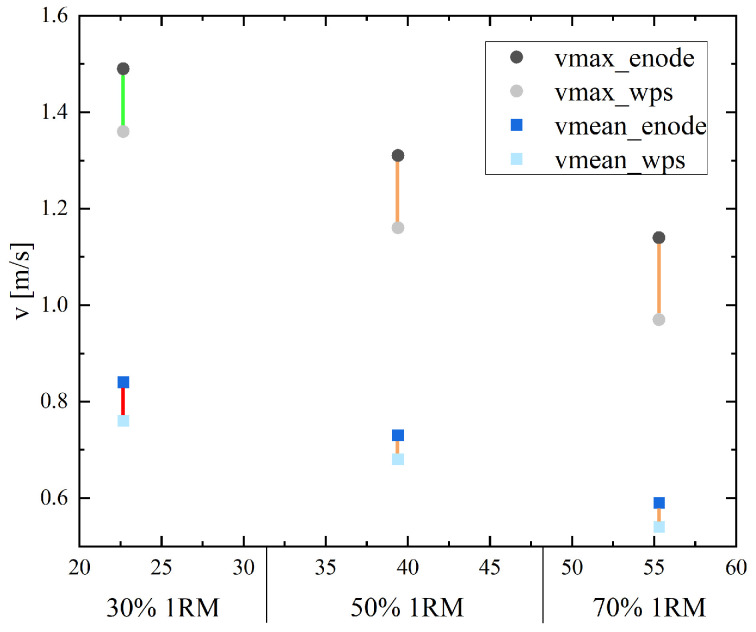
Maximum and mean velocities for the load at 30%, 50% and 70% of the 1RM for both sensors (the EnodePro and the WPS displacement sensor) during the squat measurement. The connecting line represents the size of the MAPE: MAPE < 10% green line, MAPE 10–20% orange line, MAPE > 20% red line.

Figure 6 presents the maximum and mean velocities for the load at 30%, 50% and 70% of the 1RM from both sensors during the deep squat measurement. The connecting line between the points classifies the MAPE.

**Figure 7:**
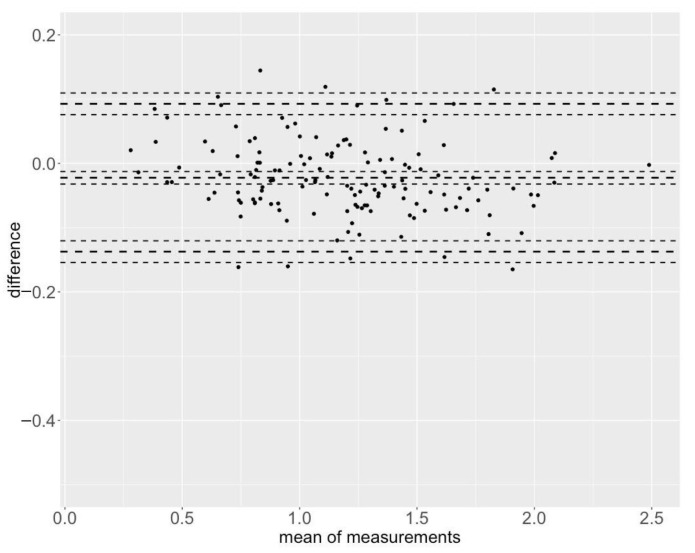
Bland–Altman plot for the maximum velocities (BP Vmax) with limits of agreement from −0.093 to 0.137 [MAE = 0.051 m/s, MAPE = 4.86%].

**Figure 8:**
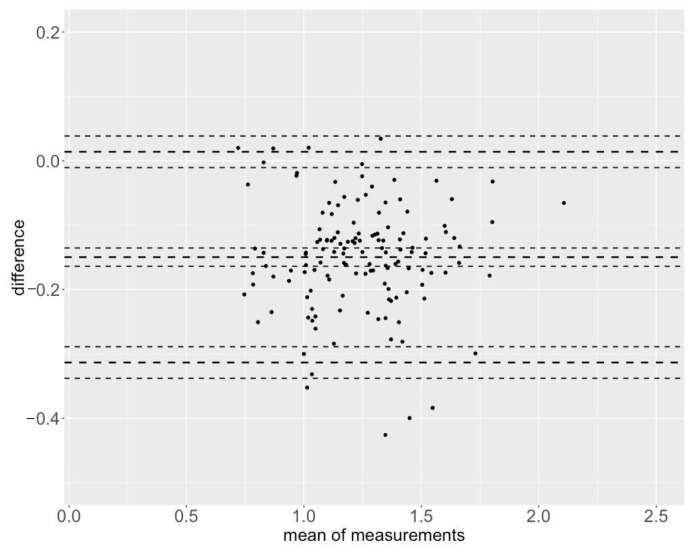
Bland–Altman plot for the maximum velocities (SQ Vmax) with limits of agreement from −0.014 to 0.313 [MAE = 0.151 m/s, MAPE = 11.71%].

The Bland–Altman plots are shown in Figure 7 and Figure 8 for the maximum velocities and in Appendix A for the mean velocities.

## 4. Discussion

The estimation of the 1RM via the inverse linear relationship between the bar velocity and the load requires, in the first step, the valid and precise measurement of the velocity. In this work, we compared the velocities measured via the EnodePro and a manual and cable-based high-precision sensor to determine the agreement among both devices within one movement. Participants performed either the bench press or the squat while both sensors were attached to the same bar. In contrast to previous studies proposing velocity sensors (e.g., the EnodePro [20,22,24]) as a valid and precise measurement device, our study indicates practically relevant measurement errors. These are indicated by absolute differences of 0.071–0.198 (MAPE = 8.28–29.90%) in the squat, while lower absolute errors in the bench press indicate movement specificity (MAE = 0.034–0.073 m/s, MAPE = 3.29–8.79%). In some measurements (BP Vmax, BP Vmean, SQ Vmax, BP V50max, BP V70max, SQ V30max, SQ V50max, SQ V70max, BP V50mean, BP V70mean), the MDC was smaller than the MAE, showing the limited validity of the MDC; this statistical parameter should provide information about the thresholds surpassed in interventional studies to attribute mean differences to the interventions rather than to measurement errors. This discrepancy in the results can be attributed to the diverse explanatory approaches, including differences in the statistical evaluation and data collection methods.

### 4.1. Statistical Issues in Velocity Sensors’ Agreement

Several studies have investigated the validity, accuracy and precision of velocity sensors in general and the EnodePro in particular. Using the free-weight back squat and the bench press, Orange et al. [34] quantified the validity of two velocity sensors at load ranges of 20% to 80% of the 1RM, with r = 0.91 and r = 0.90 for the mean and peak power at 20% 1RM. Perez-Castilla et al. [41] classified correlation coefficients ranging from 0.7 to 0.9 between two sensors as “very high to practically perfect correlations” for the validation of two velocity sensors (iLOAD and linear velocity transducer), while the authors showed both sensors’ sensitivity in tracking the mean velocity changes after resistance training, with a correlation ranging from 0.85 to 0.93 [41,42], and determined the validity of the Apple watch and the EnodePro sensor via Vicon 3D Motion capture, with r = 0.971–0.979 and r = 0.959–0.971, respectively. The authors concluded that both sensors were practically useful, with the bar-mounted Apple watch showing superior validity. Similarly, Fritschi et al. [21] validated different velocity trackers against Vicon measurements and described the Enode sensor as providing high validity, with r = 0.92–0.99. Dragutinovic and colleagues [22] also aimed to investigate the validity of the Enode sensor against a 3D MoCap and classified the sensor as highly valid, with r = 0.81–0.94 for the bench press and r = 0.62–0.74 in the squat.

Despite the questionable interpretation of the effect size magnitude of r = 0.62–0.74 as “large”, it is surprising that the frequently used statistical indices to validate velocity devices must be considered invalid if the aim is to evaluate the agreement between two velocity trackers [27,28]. When introducing new measurement devices, it is of high importance to distinguish between correlations—which describe the relationship between two parameters—and agreement between different devices. These issues were extensively discussed in previous articles, and appropriate statistical procedures were suggested. Lin [27] suggested the implementation of the concordance correlation coefficient, while Bland and Altman suggested performing measurement analyses using Bland–Altman plots [43,44,45]. These can be used to illustrate the systematic bias—for example, if one device systematically measures lower values—as well as the random measurement error [30,31]. Random measurement errors are a crucial problem in diagnostics, as they are of unknown origin and cannot be solved by adding a fixed factor. Since relative reliability indices do not account for both error sources, random as well as systematic measurement limitations must be quantified [30,31].

Although some studies have performed Bland–Altman analyses and systematic error detection [24,46], their interpretations must be critically reviewed. The classification of “acceptable validity” for typical error percentages between 5.6 and 9.2% and CVs of 5.9–11.2% [24] draws a picture of questionable comprehension regarding the practical relevance of measurement errors in performance diagnostics, considering that the sensors were attached to the exact same bar in the exact same movement. At this point, it is remarkable that the reliability values indicate even larger discrepancies for each sensor, reaching maximal values of 15.9%. It is surprising that these results led the authors recommend the sensor for practical usage. Feuerbacher et al. [46] performed BA analyses and quantified the mean differences between the measurement devices (3D MoCap, T-Force sensor and EnodePro), but the existence of significant differences was not tested. Furthermore, the results from the BA analysis were not considered when classifying the EnodePro as a valid tool (i.e., the relationship between the lower and upper limits of agreement and the reference mean were not factored in). Considering means between 0.48 and 0.97, an LoA range of −0.1–0.2 corresponds to about 30%, which could cast further doubt on their practical relevance.

Interestingly, comparable to our study, Dragutinovic et al. [22] calculated the MAPE and showed that there were task-specific random measurement errors (bench press versus squat). Despite the random measurement errors between the two devices exceeding 10%, the device’s validity was still classified as “acceptable validity for most recreational purposes” [47] when compared to validated velocity devices using 3D motion capture. The authors provided correlation coefficients (which are not a measure of agreement), quantified the CV of the typical error and calculated the overall mean bias as a percentage, as suggested by [30].

The measurement errors between different devices provide an indication of how precisely the determination of the bar velocity can be performed. Discrepancies between different devices complicate statements about the actual velocity of the bar. If, as performed in velocity-based training programs, the 1RM is estimated via imprecise velocity trackers, errors may accumulate and cause practical limitations: overestimations of the 1RM can endanger an athlete’s health and well-being, while underestimations make training stimuli ineffective. Therefore, while the previous studies’ results are not in direct contrast to ours, not only the statistical procedures (e.g., correlation coefficients) but also the interpretation of the results are complicated, as we must question the practical relevance of velocity trackers that show measurement errors of unknown variance origin of more than 10% (on average).

### 4.2. Movement Specificity: Bench Press Versus Squat

A key finding of this study is the exercise-specific accuracy of velocity measurements, which has also been observed in previous research comparing bench press and squat data. Orange et al. [34] examined the validity and reliability of a wearable inertial sensor (PUSH) compared to a linear position sensor (GymAware PowerTool). The results showed that the PUSH sensor only exhibited good validity and reliability in measuring the mean power (MP) in the bench press at 40% of the 1RM (r = 0.89; ICC = 0.83) and for squats at 20% of the 1RM for both MP (r = 0.91; ICC = 0.83) and peak power (PP) (r = 0.90; ICC = 0.80). However, the PUSH sensor failed to provide reliable estimates for other parameters at higher loads, indicating the sensor’s limited suitability for higher intensities. Interestingly, despite the similarities between the PUSH sensor and the EnodePro (both using linear accelerometers), this study reported a significantly smaller margin of error in the bench press velocity measurements (MAPE = 3.29–8.79%) compared to the squat measurements (MAPE = 8.28–29.90%). This difference may be attributed to the placement of the PUSH sensor on the forearm rather than on the barbell like the EnodePro, which provided more accurate data during the bench press than during the squat. Good agreement was observed at lower and higher velocities in the bench press (r = 0.955; ICC = 0.970 at V70max and r = 0.926; ICC = 0.956 at V30max). This could not be transferred to the squat, where better sensor agreement was observed at higher velocities and lighter loads (r = 0.941; ICC = 0.900 at V30max). Similar findings were reported by Dragutinovic et al. [22], where the EnodePro showed high validity in measuring the movement velocity (MV) during the 1RM test for both the bench press (r = 0.935) and squat (r = 0.900). However, the validity decreased at lower velocities, and the load–velocity variables and the 1RM calculations showed higher validity in the bench press (r = 0.808–0.942) than in the squat (r = 0.615–0.741), where systematic overestimations occurred.

Overall, the bench press measurements provided more consistent results between the EnodePro and Micro-Epsilon WPS sensors. In contrast, squat measurement, as a lower-body exercise, showed more variability, likely due to the increased complexity of the movement. The deeper squat position may lead to additional motion artifacts or inaccuracies in displacement measurement, especially due to the barbell’s less linear trajectory compared to the bench press. Furthermore, the sensor positioning and the dynamic forces distributed across multiple joints in the squats may have contributed to the larger discrepancies in the velocity measurements. These findings suggest that barbell velocity measurement devices may perform differently depending on the biomechanics of the movement being tested [48].

### 4.3. Determination of Velocity: Displacement Sensor Versus EnodePro

The EnodePro uses an accelerometer to measure motion by capturing acceleration (m/s^2^) along different axes. The velocity is calculated by integrating the acceleration over time, expressed mathematically as v = v_0_ +$\int a\left(t\right)dt$, where v is the velocity, v_0_ is the initial velocity and a(t) represents the acceleration at a given time. The sensor records acceleration data continuously during the movement, and its associated app (Enode App Version 2.1.1) processes and visualizes the velocity in real time. However, improper calibration or incorrect assumptions about the initial velocity (e.g., assuming that it is zero) can introduce systematic errors that propagate during integration.

In contrast, the WPS-1250-MK46-CR-P10 (Micro-Epsilon, Ortenburg, Germany) is a draw-wire sensor that directly measures the displacement and calculates the velocity by differentiating the position over time: v = $\frac{\Delta s}{\Delta t}$, where Δs is the change in position and Δt is the time interval. Movement is tracked by a retractable wire connected to a high-precision rotary encoder, which converts rotational motion into electrical signals proportional to the displacement. This method provides a high resolution and accurately detects small movements and velocities. Thus, the position of the moving object can be determined from the wire length. The accuracy of the velocity measurement depends on the encoder’s resolution and the sensor’s sampling rate. The finer the displacement changes can be detected and the faster the data are collected, the more accurately the velocity can be calculated. The WPS-1250-MK46-CR-P10 offers a high resolution; however, both methods can be prone to errors from drift or noise in the signal processing.

### 4.4. Practical Applications and Implications

The findings of this study provide several practical implications for coaches, sport scientists and athletes, but individual variations should not be overlooked. While the agreement between the EnodePro and the high-resolution manual sensor was acceptable when focusing on mean-based statistics and the relative reliability in the bench press, this study highlights the variance in previous studies’ results, showing large estimation errors in the squat. While previous articles [13,23] partly attributed the practically relevant accuracy limitations of 1RM estimations to linear regression models, this study highlights the more fundamental problems of this monitoring approach, as it seems that the sensor is not able to measure the velocity with the required precision. Deriving training recommendations from velocity profiles measured via the EnodePro must be considered infeasible.

Although the overall reliability statistics suggested better agreement in the results for the bench press, practitioners and coaches must be aware of the accompanying random measurement errors. For the 70%1RM bar velocity, there was still an MAPE of about 8%. In top athletes that can bench-press 200 kg, this would still result in an over- or underestimation of 16 kg. The acceptance of such measurement errors in exercise and training planning is the coaches’ and athletes’ individual choice.

Therefore, with our extended statistical agreement analysis, we wish to raise awareness about the different aspects of the measurement errors, which can meaningfully affect practical settings and, when the maximal strength is overestimated, harm athletes’ health. We call for caution when applying the EnodePro, especially when seeking to control loads in deep squats.

Accelerometers like the EnodePro have potential for 1RM estimation, aiding training regulation and planning. However, the current methods often rely on fixed-percentage loads based on a previously determined 1RM. Given the inconsistent reliability of velocity profiles in squats, as noted in previous studies [49], it is recommended to continue using traditional methods to determine the maximal strength [13], as conventional 1RM tests have been shown to be reliable [2]. Before velocity-based approaches can be widely adopted, further research is necessary to develop reliable estimation protocols.

### 4.5. Limitations

Despite the strengths of this study, some limitations should be acknowledged. Although the sample size was sufficient for statistical analysis, the participants varied widely in their training experience. This may have influenced their movement execution and contributed to measurement variability. Future studies should categorize participants according to their training experience to better isolate the effects of movement competence on the measurement accuracy.

The squat depth was not controlled using video analysis software, which may have led to variations in depth. Implementing such controls could improve the consistency in future studies. Furthermore, the velocity measurements in this study were only performed at three load levels (30%, 50% and 70% of 1RM), which may not fully capture the entire range of velocities encountered in strength training. Additionally, the 1RM was determined in a pre-test, but it can vary depending on the participants’ physical condition on the test day. This variability could introduce bias when calculating the percentages of the 1RM during data collection.

At this point, it must be noted that the performed squatting movement was executed in a Smith machine. The free-weight straight bar squat could show significantly different results, with reasonably assumed higher variations in the movement patterns.

In the present investigation, the data were recorded independently by each system and later combined in Excel spreadsheets, which could have led to extraction errors. There was no synchronization between these devices during data recording, which may have introduced some variability into the results. Additionally, the EnodePro was attached centrally to the barbell via its magnetic backside according to standard protocols, while the displacement sensor was positioned at one end. This divergent positioning might have caused minor discrepancies in the collected measurement data, as suggested by previous studies [21].

The data processing methods used by the Enode App are not fully transparent. Differences in how the mean velocities are calculated, as well as the use of smoothing filters (e.g., Kalman filters or low-pass filters), may affect the accuracy. However, if these filters are applied too aggressively, they may also smooth out real changes in acceleration, thereby reducing the accuracy of the velocity measurements. Furthermore, differences in the sampling rates may also influence the accuracy. The EnodePro records at 200 Hz, while the displacement sensor sampled at 1000 Hz, potentially yielding more accurate results for the displacement sensor.

## 5. Conclusions

This study revealed an unexpected measurement error between the testing devices. Since both velocity devices were attached to the same bar (but in different positions), a minimal testing error was assumed. The agreement ICCs, ranging from low to high, and the concordance correlation coefficients between 0.110 and 0.989, were accompanied by MAEs ranging from 0.036 m/s to 0.169 m/s (MAPE = 4.09–42.15%). The reliability statistics raise potential concerns about its practical applicability. The EnodePro slightly overestimates the velocities of the WPS, whereas overestimation becomes more pronounced at the mean velocities and persists across all data partitions. Specifically, at higher velocities (V30max, V30mean), the overestimation appears to be greater (see Table 2 and Table 3). Overall, the deep squat analysis results suggest greater divergence between the two sensors. This could indicate that the complex movement pattern and potential instability during squatting affected the consistency of the velocity measurements. The results raise doubt about the precision and accuracy of the EnodePro, especially for complex movements. Practitioners are advised to prohibit the use of these devices until novel techniques with optimized (in this case, maximized) validity are available, as accurate training control is hindered by precision limitations.

## Figures and Tables

**Table 1:** Maximum and mean velocities for the bench press (BP) and deep squat (SQ) measurements, including means and standard deviations, as well as statistics from the reliability and agreement analyses.

Parameter	M ± SD EnodePro in m/s	M ± SD WPS in m/s	Pearson’s r	ICC (95% CI)	Concordance r	MAE in m/s	MPE in %	MAPE in %	Mean Diff. (*p*-Value)	SEM	MDC	Limits of Agreement (95% CI)
BP: Vmax	1.19 ± 0.43	1.18 ± 0.42	0.991	0.995 (0.991–0.997)	0.989 (0.985–0.992)	0.051	−1.77	4.86	0.022 (*p* < 0.001)	0.004	0.012	−0.093–0.137
BP: Vmean	0.73 ± 0.23	0.68 ± 0.22	0.987	0.984 (0.793–0.995)	0.968 (0.957–0.976)	0.048	−6.39	7.31	0.043 (*p* < 0.001)	0.005	0.012	−0.029–0.115
SQ: Vmax	1.31 ± 0.25	1.16 ± 0.25	0.945	0.892 (−0.109–0.971)	0.803 (0.752–0.845)	0.151	−13.75	11.71	0.150 (*p* < 0.001)	0.027	0.076	−0.014–0.313
SQ: Vmean	0.72 ± 0.18	0.66 ± 0.17	0.668	0.772 (0.623–0.855)	0.628 (0.521–0.715)	0.095	−18.24	23.49	0.060 (*p* < 0.001)	0.068	0.188	−0.219–0.339

BP—bench press; SQ—squat; Vmax—maximum velocity; Vmean—mean velocity; M—mean value; SD—standard division; ICC—intraclass coefficient for two-way agreement; MAE—mean absolute error; MPE—mean percentage error; MAPE—mean absolute percentage error; SEM—standard error of measurement; MDC—minimal detectable change.

**Table 2:** Maximum velocities sorted by 30%, 50%, 70% of 1RM for the bench press (BP) and deep squat (SQ) measurements, including means and standard deviations, as well as statistics from the reliability and agreement analyses.

Parameter	M ± SD EnodePro in m/s	M ± SD WPS in m/s	Pearson’s r	ICC (95% CI)	Concordance r	MAE in m/s	MPE in %	MAPE in %	Mean Diff. (*p*-Value)	SEM	MDC	Limits of Agreement (95% CI)
BP: V30max	1.63 ± 0.35	1.59 ± 0.32	0.926	0.956 (0.918–0.976)	0.914 (0.855–0.949)	0.065	−8.36	4.09	0.044 (*p* = 0.021)	0.028	0.077	−0.217–0.305
BP: V50max	1.18 ± 0.27	1.16 ± 0.25	0.979	0.987 (0.975–0.993)	0.973 (0.955–0.984)	0.049	−1.11	4.33	0.018 (*p* = 0.025)	0.006	0.018	−0.093–0.129
BP: V70max	0.78 ± 0.23	0.77 ± 0.19	0.955	0.970 (0.946–0.983)	0.941 (0.902–0.964)	0.058	−1.81	7.94	0.019 (*p* = 0.053)	0.013	0.036	−0.119–0.157
SQ: V30max	1.49 ± 0.24	1.36 ± 0.23	0.941	0.900 (−0.061–0.974)	0.815 (0.718–0.882)	0.131	−9.78	9.99	0.129 (*p* < 0.001)	0.026	0.071	−0.030–0.288
SQ: V50max	1.31 ± 0.20	1.16 ± 0.18	0.918	0.822 (−0.178–0.953)	0.693 (0.569–0.786)	0.151	−13.11	13.32	0.150 (*p* < 0.001)	0.034	0.094	−0.007–0.307
SQ: V70max	1.14 ± 0.19	0.97 ± 0.18	0.895	0.772 (−0.187–0.939)	0.624 (0.489–0.729)	0.169	−18.19	18.19	0.170 (*p* < 0.001)	0.018	0.048	0.001–0.338

BP—bench press; SQ—squat; Vmax—maximum velocity; M—mean value; SD—standard division; ICC—intraclass coefficient for two-way agreement; MAE—mean absolute error; MPE—mean percentage error; MAPE—mean absolute percentage error; SEM—standard error of measurement; MDC—minimal detectable change.

**Table 3:** Mean velocities sorted by 30%, 50%, 70% of 1RM for the bench press (BP) and deep squat (SQ) measurements, including means and standard deviations, as well as statistics from the reliability and agreement analyses.

Parameter	M ± SD EnodePro in m/s	M ± SD WPS in m/s	Pearson’s r	ICC (95% CI)	Concordance r	MAE in m/s	MPE in %	MAPE in %	Mean Diff. (*p*-Value)	SEM	MDC	Limits of Agreement (95% CI)
BP: V30mean	0.94 ± 0.19	0.88 ± 0.18	0.882	0.904 (0.644–0.961)	0.822 (0.717–0.890)	0.073	8.36	8.79	−0.068 (*p* < 0.001)	0.028	0.078	−0.109–0.246
BP: V50mean	0.73 ± 0.15	0.68 ± 0.14	0.971	0.957 (0.378–0.988)	0.916 (0.867–0.948)	0.053	−3.84	8.08	0.048 (*p* < 0.001)	0.007	0.021	−0.022–0.119
BP: V70mean	0.50 ± 0.14	0.48 ± 0.12	0.969	0.972 (0.887–0.989)	0.944 (0.907–0.966)	0.036	−5.15	7.17	0.026 (*p* < 0.001)	0.006	0.016	−0.043–0.095
SQ: V30mean	0.84 ± 0.16	0.76 ± 0.19	0.445	0.580 (0.223–0.772)	0.403 (0.151–0.605)	0.123	−36.18	42.15	0.129 (*p* < 0.001)	0.123	0.341	−0.297–0.447
SQ: V50mean	0.73 ± 0.14	0.68 ± 0.11	0.788	0.835 (0.591–0.922)	0.712 (0.558–0.819)	0.076	−7.44	11.92	0.049 (*p* < 0.001)	0.034	0.094	−0.115–0.212
SQ: V70mean	0.59 ± 0.16	0.54 ± 0.10	0.511	0.604 (0.264–0.784)	0.427 (0.209–0.605)	0.086	−11.89	17.24	0.058 (*p* = 0.007)	0.038	0.103	−0.213–0.328

BP—bench press; SQ—squat; Vmean—mean velocity; M—mean value; SD—standard division; ICC—intraclass coefficient for two-way agreement; MAE—mean absolute error; MPE—mean percentage error; MAPE—mean absolute percentage error; SEM—standard error of measurement; MDC—minimal detectable change.

**Figure A1:**
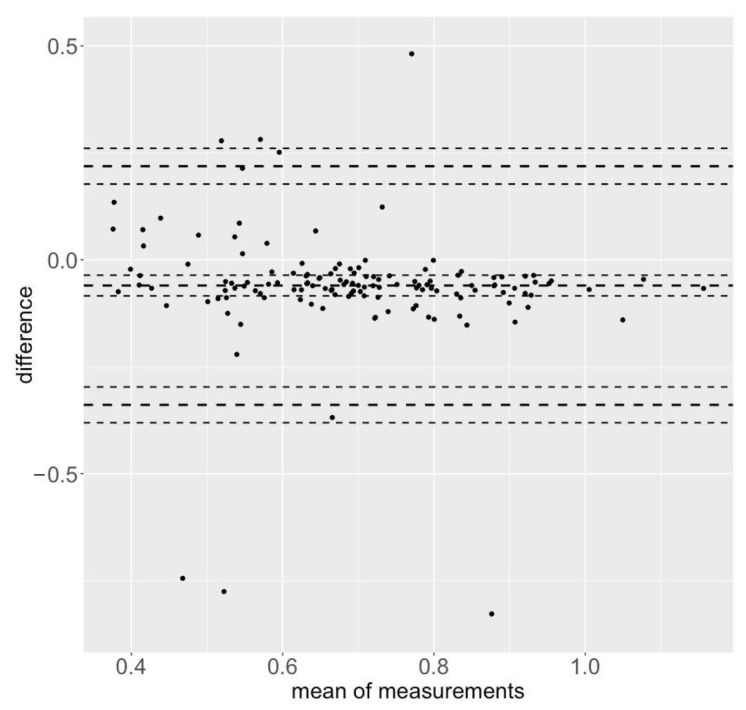
Bland–Altman plot for the mean velocities (SQ Vmean) with limits of agreement from −0.219 to 0.339 [MAE = 0.095 m/s, MAPE = 23.49%].

**Figure A2:**
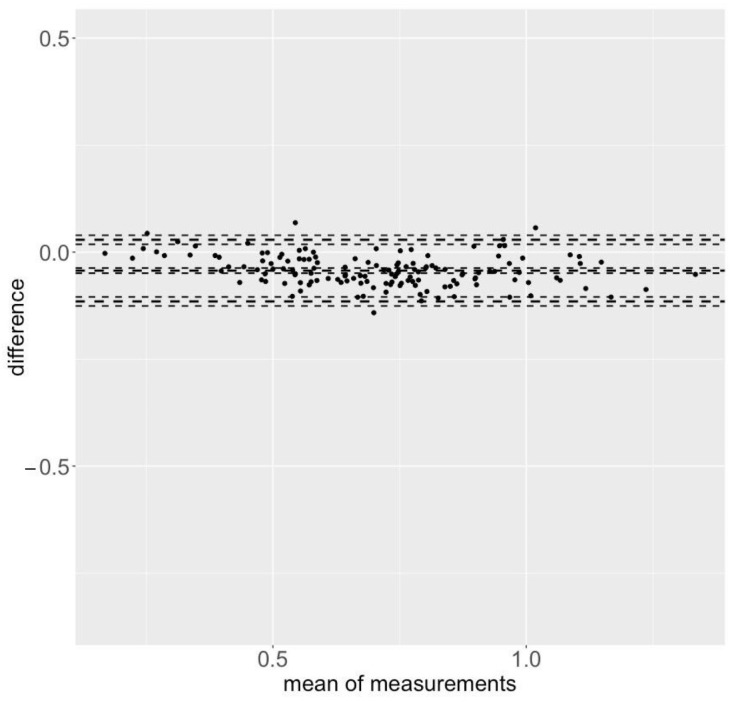
Bland–Altman plot for the mean velocities (BP Vmean) with limits of agreement from −0.029 to 0.115 [MAE = 0.048; MAPE = 7.31%].

## Data Availability

Original data can be obtained from the corresponding author upon reasonable request.

## Appendix A. Statistical Analysis with Outliers Included

**Table A1:** Maximum and mean velocities for the bench press (BP) and deep squat (SQ) measurements, including means and standard deviations, as well as statistics from the reliability and agreement analyses (Analyses includes outliers).

Parameter	M ± SD EnodePro in m/s	M ± SD WPS in m/s	Pearson’s r	ICC (95% CI)	Concordance r	MAE in m/s	MPE in %	MAPE in %	Mean Diff. (*p*-Value)	SEM	MDC	Limits of Agreement (95% CI)
BP: Vmax	1.19 ± 0.45	1.17 ± 0.42	0.965	0.981	0.963	0.067	−0.79	6.28	0.015 (*p* = 0.135)	0.016	0.045	−0.218–0.248
BP: Vmean	0.72 ± 0.24	0.68 ± 0.22	0.929	0.954	0.911	0.059	−5.72	8.66	0.039 (*p* < 0.001)	0.019	0.054	−0.139–0.217
SQ: Vmax	1.31 ± 0.26	1.16 ± 0.25	0.914	0.876	0.778	0.157	−14.06	14.69	0.151 (*p* < 0.001)	0.038	0.104	−0.058–0.360
SQ: Vmean	0.72 ± 0.18	0.66 ± 0.17	0.656	0.763	0.615	0.097	−18.37	23.87	0.061 (*p* < 0.001)	0.071	0.196	−0.223–0.345

BP—bench press; SQ—squat; Vmax—maximum velocity; Vmean—mean velocity; M—mean value; SD—standard division; ICC—intraclass coefficient for two-way agreement; MAE—mean absolute error; MPE—mean percentage error; MAPE—mean absolute percentage error; SEM—standard error of measurement; MDC—minimal detectable change.

**Table A2:** Maximum velocities sorted by 30%, 50%, 70% of 1RM for the bench press (BP) and deep squat (SQ) measurements, including means and standard deviations, as well as statistics from the reliability and agreement analyses (Analyses includes outliers).

Parameter	M ± SD EnodePro in m/s	M ± SD WPS in m/s	Pearson’s r	ICC (95% CI)	Concordance r	MAE in m/s	MPE in %	MAPE in %	Mean Diff. (*p*-Value)	SEM	MDC	Limits of Agreement (95% CI)
BP: V30max	1.62 ± 0.35	1.59 ± 0.32	0.884	0.935	0.876	0.077	−1.93	4.70	0.029 (*p* = 0.210)	0.042	0.117	−0.294–0.353
BP: V50max	1.17 ± 0.27	1.16 ± 0.25	0.948	0.971	0.943	0.058	−0.10	5.13	0.006 (*p* = 0.641)	0.015	0.041	−0.166–0.178
BP: V70max	0.77 ± 0.23	0.76 ± 0.19	0.930	0.956	0.914	0.065	−0.36	9.01	0.008 (*p* = 0.506)	0.018	0.051	−0.163–0.179
SQ: V30max	1.47 ± 0.27	1.33 ± 0.25	0.853	0.859	0.748	0.151	−11.02	12.76	−0.135 (*p* < 0.001)	0.054	0.149	−0.145–0.416
SQ: V50max	1.31 ± 0.20	1.16 ± 0.18	0.918	0.822	0.693	0.151	−13.11	13.32	0.150 (*p* < 0.001)	0.037	0.102	−0.007–0.307
SQ: V70max	1.14 ± 0.19	0.97 ± 0.18	0.895	0.772	0.624	0.169	−18.19	18.19	0.170 (*p* < 0.001)	0.041	0.114	0.001–0.338

BP—bench press; SQ—squat; Vmax—maximum velocity; M—mean value; SD—standard division; ICC—intraclass coefficient for two-way agreement; MAE—mean absolute error; MPE—mean percentage error; MAPE—mean absolute percentage error; SEM—standard error of measurement; MDC—minimal detectable change.

**Table A3:** Mean velocities sorted by 30%, 50%, 70% of 1RM for the bench press (BP) and deep squat (SQ) measurements, including means and standard deviations, as well as statistics from the reliability and agreement analyses (Analyses includes outliers).

Parameter	M ± SD EnodePro in m/s	M ± SD WPS in m/s	Pearson’s r	ICC (95 % CI)	Concordance r	MAE in m/s	MPE in %	MAPE in %	Mean Diff. (*p*-Value)	SEM	MDC	Limits of Agreement (95 % CI)
BP: V30mean	0.93 ± 0.21	0.88 ± 0.18	0.737	0.827	0.701	0.086	−6.84	9.97	0.053 (*p* = 0.012)	0.059	0.165	−0.227–0.333
BP: V50mean	0.72 ± 0.15	0.68 ± 0.14	0.949	0.950	0.904	0.055	−6.12	8.37	0.043 (*p* < 0.001)	0.019	0.055	−0.052–0.139
BP: V70mean	0.49 ± 0.14	0.48 ± 0.12	0.955	0.967	0.934	0.037	−4.19	7.64	0.022 (*p* < 0.001)	0.011	0.029	−0.060–0.103
SQ: V30mean	0.83 ± 0.17	0.75 ± 0.19	0.437	0.574	0.397	0.131	−36.46	43.30	0.078 (*p* = 0.010)	0.128	0.354	−0.305–0.462
SQ: V50mean	0.73 ± 0.14	0.68 ± 0.11	0.788	0.835	0.712	0.076	−7.44	11.92	0.049 (*p* < 0.001)	0.034	0.094	−0.115–0.212
SQ: V70mean	0.59 ± 0.16	0.54 ± 0.10	0.511	0.604	0.427	0.086	−11.89	17.24	0.058 (*p* = 0.007)	0.087	0.241	−0.213–0.328

BP—bench press; SQ—squat; Vmean—mean velocity; M—mean value; SD—standard division; ICC—intraclass coefficient for two-way agreement; MAE—mean absolute error; MPE—mean percentage error; MAPE—mean absolute percentage error; SEM—standard error of measurement; MDC—minimal detectable change.
